# Socioeconomic Impact of Foot and Mouth Disease Outbreaks on Smallholder Cattle Farmers in Yogyakarta, Indonesia

**DOI:** 10.3390/vetsci12060542

**Published:** 2025-06-03

**Authors:** Agung Triatmojo, Budi Guntoro, Péter Strausz, Mujtahidah Anggriani Ummul Muzayyanah, Robi Agustiar, Szilvia Kusza

**Affiliations:** 1 Department of Livestock Socioeconomics, Faculty of Animal Science, Universitas Gadjah Mada, Yogyakarta 55281, Indonesia; budiguntoro@ugm.ac.id (B.G.); m_anggriani_um@ugm.ac.id (M.A.U.M.); robiagustiar@mail.ugm.ac.id (R.A.); 2 Department of Management, Institute of Strategy and Management, Corvinus University of Budapest, 1093 Budapest, Hungary; peter.strausz@uni-corvinus.hu; 3 Centre for Agricultural Genomics and Biotechnology, Faculty of Agricultural and Food Sciences and Environmental Management, University of Debrecen, 4032 Debrecen, Hungary

**Keywords:** foot and mouth disease, KAP, cattle farmer, treatment cost

## Abstract

Foot and Mouth Disease (FMD) poses significant challenges to livestock management and agricultural economies worldwide. This study examines the effect of farmers’ sociodemographic factors on livestock infected with Foot and Mouth Disease (FMD) and analyzes its socioeconomic impact on smallholder farmers in Indonesia. This study collected data from 992 households (202 infected and 790 non-infected) in the special region of Yogyakarta province. The research used propensity score matching (PSM) treatment effect analysis to assess the socioeconomic impact of FMD outbreaks on smallholder farmers. Our results demonstrated that FMD significantly increased (*p *< 0.01) smallholder farmers’ social behavior, including knowledge, attitude, and practice (KAP). Furthermore, farmers whose animals are already infected with FMD must spend an additional IDR 258,000 to IDR 270,000 on treatment compared to non-infected ones. This study provides empirical evidence that farmer characteristics, including women’s decision-making, income, farming group, and cattle ownership, determine the likelihood of FMD infection, which implies that farmers with specific characteristics may heighten the risk of FMD infection. We concluded that FMD has changed social behavior and accelerated economic loss for smallholder farmers. Hence, farmers with animals at risk of FMD infection are prioritized in FMD control programs.

## Simple Summary

This study investigates how farmers’ social and demographic factors influence the spread of Foot and Mouth Disease (FMD) in livestock and its economic impact on smallholder farmers in Indonesia. The goal was to assess the effects of these factors on FMD infection and the financial strain it places on farmers. The findings revealed that FMD altered farmers’ behaviors regarding animal health and increased their treatment costs. This study concluded that factors like economic situation, decision-making roles, and cattle ownership heightened the risk of FMD. It emphasizes the importance of prioritizing high-risk farmers in FMD control efforts to reduce both social and economic burdens.

## 1. Introduction

**Figure 1:**
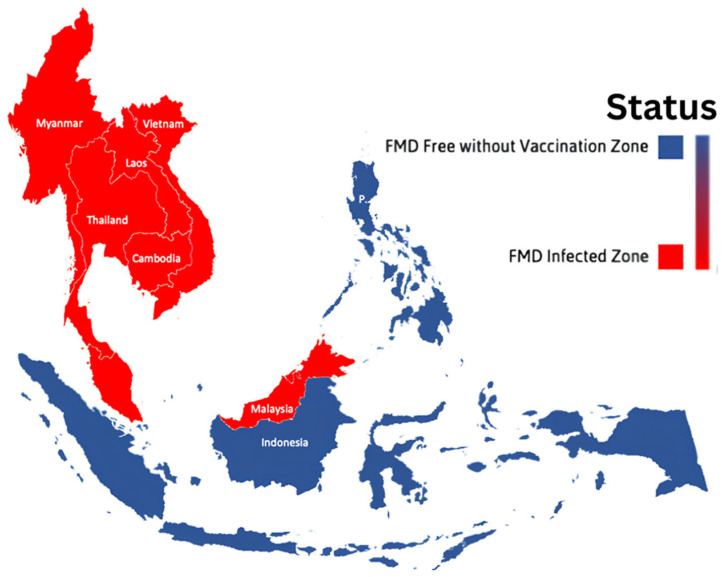
The distribution of Foot and Mouth Disease (FMD) occurrences throughout southeast Asia.

One of the most socially and economically devastating diseases in the world’s livestock industry is Foot and Mouth Disease (FMD). Southeast Asia (SEA) is one of the regions impacted by the FMD outbreak [1,2]. FMD is endemic to the continental SEA (Vietnam, Myanmar, Thailand, Laos, Peninsular Malaysia, and Cambodia); currently, SEA countries that are free from FMD without the need for vaccination include Brunei Darussalam, Indonesia, Singapore, and the Philippines (Figure 1) [3].

**Figure 2:**
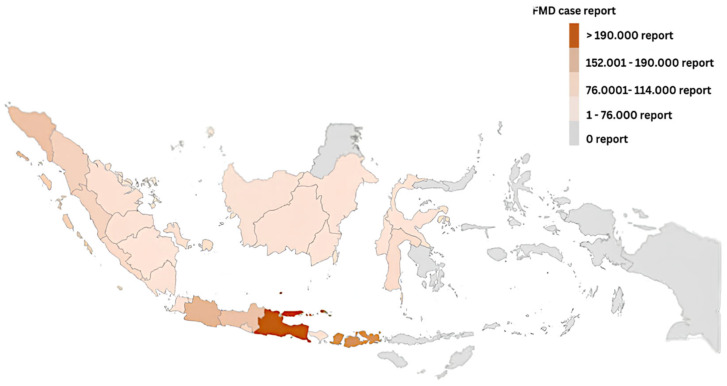
A map of Indonesia’s FMD-affected areas as of 3 November 2022.

Nevertheless, an FMD outbreak occurred in cattle on 12 April 2022, in East Java province, Indonesia, which was caused by an Aphthovirus species (family *Picornaviridae*). On 6 May 2022, the World Organization of Animal Health announced the confirmation of 3496 cases in the provinces of Aceh and East Java, suspending Indonesia’s FMD-free designation as of 12 April 2022 [1,4,5]. As of 3 November 2022, there have been 570,137 cases (morbidity rate = 1.04 percent) among 54,767,135 cattle, 9785 deaths (morbidity rate = 0.02 percent), 12,650 culls, and 5,199,595 vaccinations (Figure 2). FMD primarily affects large ruminants, particularly cattle [6,7]. Following data from [8], 94.27 percent of cattle were infected, including buffalo (4.56 percent), goats (0.80 percent), sheep (0.36 percent), and swine (0.02 percent). Large ruminants are the focus of intensive eradication since they have an enormous prevalence of FMD cases and the highest economic value.

In addition to the re-emergence of FMD in Southeast Asia, the year 2025 marked a significant shift in the global epidemiological landscape of the disease, with confirmed outbreaks reported in several European countries. This development underscores the need for renewed international vigilance and coordinated control efforts, as FMD is no longer confined to traditionally endemic regions. The virus has been detected in areas of Europe typically free from FMD, with Germany recently reporting an outbreak, although it is now FMD-free. However, outbreaks continue in Hungary and Slovakia. In response, the UK has banned imports of meat and dairy products from affected European countries, including Austria, due to the outbreak in Hungary [9].

Historically, FMD was first identified in Indonesia in September 1887 (East Java, Malang), 1892 (Sumatra, East Java), 1902 (Sulawesi), 1906 (Madura, Kalimantan), 1907 (Sulawesi), 1911 (West Nusa Tenggara), 1913 (Madura), 1952, 1956–1958, and 1962 (Bali), 1972–1974, and last in 1983, according to the World Reference Laboratory for Foot and Mouth Disease website http://www.wrlfmd.org (accessed on 12 August 2024). The Indonesian livestock authorities conducted control programs for smallholder farmers to eradicate FMD. These programs included stamping out, controlling livestock movements, disinfecting vehicles, closing infected areas to prevent livestock movement, controlling abattoirs including meat distribution and slaughter, controlling quarantine stations, isolating as well as treating livestock, disinfecting livestock premises, mass vaccinating, and reporting cases. Thus, Indonesia had been declared FMD-free since 1986 [10,11].

Despite a period of almost forty years without a single outbreak of Foot and Mouth Disease, Indonesia was unable to avoid the occurrence of the ailment. The suspected recurrence of FMD can be attributed to (1) inadequate border surveillance. Indonesia, the most extensive archipelagic nation globally (Figure 2), possesses an exceptionally lengthy coastline [12] and (2) poor livestock management practices among smallholder farmers. Nevertheless, smallholder farmers in Indonesia provide more than 90% of the country’s domestic cattle production [13,14,15]; however, the behavior of smallholder farmers is influenced by obsolete knowledge and attitudes [16]. Poor disease knowledge and attitudes associated with disease prevalence can propel underreporting and awareness deficits [17]. Bandura [18] argued that the behaviors of people are shaped by the surrounding society. Therefore, the increase in FMD in Indonesia is presumed to lead to societal changes in farmer behavior, including knowledge, attitudes, and livestock management practices.

Concurrently, the veterinary sector incurs significant financial losses due to productivity declines caused by FMD. The effects refer to the direct negative consequences that hinder livestock production, including reduced weight gains, decreased fertility, and increased mortality, particularly in young animals. Additionally, there are associated costs related to addressing diseases or infections, such as expenses for treatments, vaccinations, movement restrictions, and eradication precautions [19]. Moreover, the primary infectious FMD poses a severe risk to ruminants. Although adult animal mortality is typically low, millions of cattle have been slaughtered in efforts to quickly control and eradicate FMD [20,21,22]. Recent research on the impact of FMD on smallholder farmers has been reported. According to studies conducted in Cambodia, an outbreak of FMD was followed by a loss of 54–92% of the value of animals and a 4.4–11.7% annual decline in household income [23,24]. It corresponds with the FMD-related loss of 22–30% in animal values observed in Laos [25].

Presently, there are insufficient data regarding the societal and financial ramifications of FMD in Indonesia. Moreover, the specific farmer factors that make their livestock more susceptible to FMD remain unidentified. Therefore, this work aspires to fill this gap and aims to examine the farmer characteristics that determine FMD prevalence and analyze the socioeconomic impact of FMD on smallholder cattle farmers. This study provides insights into the behavior alterations in livestock management programs that farmers conduct in response to FMD and estimates of the additional costs that farmers should incur during FMD outbreaks.

## 2. Materials and Methods

### 2.1. Data Collection

This study is a survey implementing a cross-sectional design. One-to-one interviews were conducted using a predesigned structured questionnaire. A stratified random sampling technique selected smallholder farmers from four DIY regencies (Gunungkidul, Bantul, Kulon Progo, and Sleman). A farmer who typically possesses three heads of cattle with less than 5000 m^2^ of land, alongside an average of four family members, is considered a smallholder [26]. All farmers involved in this study focus on production primarily for domestic consumption. The special region of Yogyakarta province on Java Island was chosen as the study area because more than half of the farmers were affected by the FMD outbreak.

**Figure 3:**
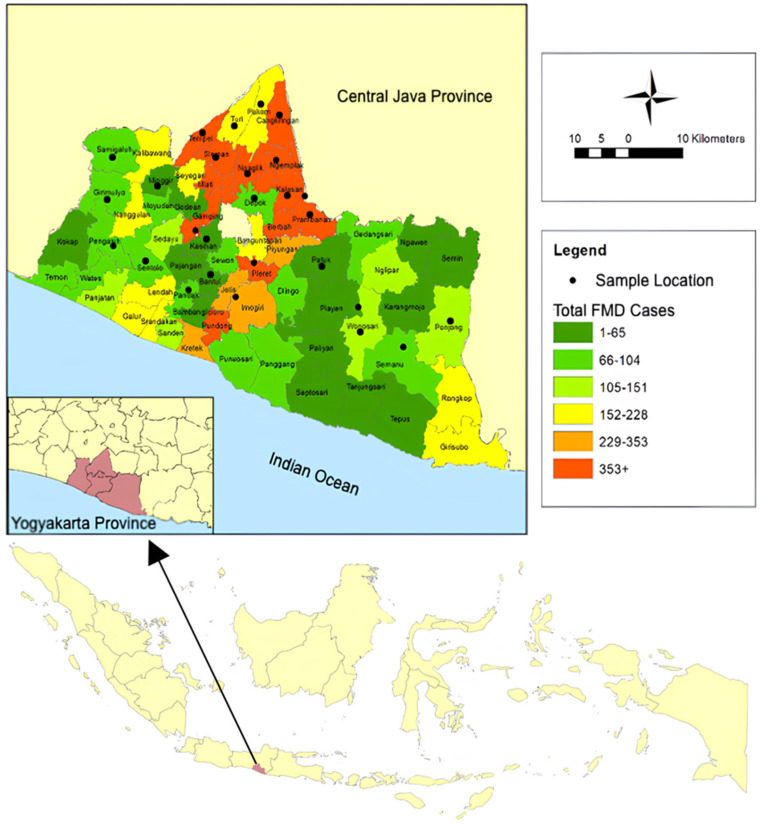
FMD cases and the location of the respondent sample.

Based on the higher cattle population, survey data on cattle farming were collected from each district of every regency. Therefore, areas with higher cattle numbers were surveyed with more respondents. However, there are fewer cases of FMD in the central population (Gunungkidul) due to the assumption that the type of cattle rearing is individual rather than communal (Sleman). Thus, in areas with more FMD cases, more samples were taken with fewer respondents, with 2 to 3 respondents per group of farmers. Figure 3 and Table 1 illustrate the respondents’ distribution by district for each regency. We ensured the representativeness of the sample by considering regional distribution and farmer characteristics. Despite these efforts, potential selection bias could still exist, as certain thresholds and requirements were applied in selecting participants. However, the high number of respondents in this study helped mitigate any such biases, enhancing the robustness of the findings.

In addition, the Yogyakarta area serves as a livestock traffic area and gateway for cattle trade [27]. The survey was conducted between September and October 2022. A total of 992 respondents were surveyed for the main data who were then categorized into groups according to their exposure to the FMD outbreak (FMD-infected or non-infected animals). The response rate for this study was 99%, with 992 out of 1000 initially contacted farmers completing the survey. The data were grouped into infected and non-infected categories during the pandemic of FMD. The respondents possessed the required permission and were informed regarding the research objective. If the respondents agreed to be interviewed, they were requested to sign the consent letter. The respondents were assured that their information will be kept confidential and used only for research.

### 2.2. Variable Measurement

The distinct composite variables covered the farmers’ characteristics, farm-associated information, and farmers’ knowledge, attitudes, and practices (Table 2). This study used the KAP model that originates in learning theory [18], as adopted by [28,29]. Knowledge, attitudes, and practices are significant elements in models for behavioral change. Knowledge is the cognitive and non-symbolic experience of meaning that results from information understanding [30,31]. Attitude indicates a positive or negative assessment of an issue [32]. Practice refers to frequent behaviors impacted by broadly recognized social norms and values [33].

A knowledge assessment was conducted using a score system consisting of eleven questions related to the disease, prevention, and treatment of FMD. According to [35], the objective knowledge index was established by assigning a score of 1 to each true or false statement answered correctly by the respondent, and a score of 0 to erroneous responses. The social outcomes were then summed and divided into two categories: “less” has a total score of (1–5), and “good” (6–11). A total of six questions were included to assess farmers’ responses to FMD outbreaks, using a 6-point Likert scale to measure their attitudes. A farmer’s attitude was classified as “negative” if a rating (1–3) was given and “positive” otherwise (4–6). Furthermore, equivalent to knowledge, practice is measured using a scoring method with nine behavioral assertions of appropriate disease management programs. Farmers were marked as “less” if they accomplished fewer than four activities and as “good” if they conducted six to nine. The categories “less” and “negative” were then encoded as 0, whereas “good” and “positive” were encoded as 1. Hence, the total KAP ratings were calculated as a variable social impact.

### 2.3. Statistical Analysis

The statistical analysis in this study was conducted using STATA 14, with the “psmatch2” package. Continuous variables, including farmer characteristics, were compared using nonparametric Wilcoxon tests. To analyze the outcomes, we implemented an econometric model using the Average Treatment on the Treated (ATT) approach, based on the propensity score matching (PSM) method [36,37,38,39,40,41,42]. PSM is particularly advantageous in observational studies because it reduces confounding bias by balancing observed covariates between treatment and control groups, making them more comparable—similar to what randomization achieves in experimental studies [43].

First, propensity scores were calculated using a multivariable logistic regression model. The dependent variable in this model was the group classification (FMD-infected vs. non-infected), and the independent variables were baseline farmer characteristics that were unbalanced between groups, including age, education, household size, land size, women’s involvement in decision-making, income, farmer group, cattle ownership, farming system, and farming experience.

Second, to ensure the propensity scores were within the common support area, we excluded outliers based on the score distribution of both groups. A matching test was then performed to confirm that the averages of each covariate and the propensity score did not significantly differ between the treated and untreated groups [42]. As noted in prior research [26], this step prevents bias that could arise from linking distinct treatment groups (infected vs. non-infected) and ensures more accurate matching.

The third step involved matching farmers with FMD-infected animals to farmers with non-infected animals based on their propensity scores, which represent the likelihood of FMD infection given their characteristics [40,42]. Finally, the mean outcome difference between the two paired groups was calculated to estimate the social and economic impacts of FMD outbreaks on smallholder farmers. In addition, the ATT values derived through PSM also enabled us to assess behavioral changes in livestock management practices using the KAP (Knowledge, Attitude, and Practice) score and estimate additional cost differences between treated and untreated groups due to the FMD outbreak [40,42].

Rosenbaum and Rubin [40] first introduced the concept of the propensity score, defined as the likelihood of receiving treatment given the pre-treatment parameters. The treatment impact analysis based on PSM for FMD can be mathematically expressed as follows:

(1) $$p\;\left(X\right)\equiv \mathrm{Pr}\;\left\{D=1\;\right|X\}=E\;\left\{D\;\right|\;X\}$$

where *D* = {1, 0} is a binary treatment indicator, 1 represents farmers with FMD-infected animals (treated group), and 0 represents farmers without FMD-infected animals (control group). The variable *X* denotes the multidimensional vector of pre-treatment characteristics. If the treatment assignment is randomized within cells defined by *X*, then it is also randomized within cells defined by the propensity score *p(X)* [40]. Consequently, assuming a population of units represented by *i*, the average treatment effect on the treated (ATT) can be estimated as follows:

(2) $${ATT}_{PSM}\equiv E\left\{E\left\{Y_{1i}\right|D_{i}=1,\;p\;\left(X_{i}\right)\right\}-\left\{E\left\{Y_{0i}\right|D_{i}=0,\;p\;\left(X_{i}\right)\right\}\;|\;D_{i}=1\}$$

where *Y*_1*i*_ represents the outcome if farmer iii’s animal was infected by FMD, and *Y*_0_*_i_* represents the outcome if their animal was not infected. The ATT measures the difference in outcomes between treated and control groups, calculated from the distribution of participants’ propensity scores, assuming conditional independence and sufficient overlap in *p(X_i_)*. To avoid sampling bias, we discard observations where the propensity scores fall outside the common support range, i.e., the highest propensity score in the treated group and the lowest propensity score in the control group. Matching estimates are only valid if matching tests confirm that the propensity score distributions of both groups are comparable and that the average of each covariate and propensity score does not significantly differ between groups. If the common support condition is met, matching can proceed without introducing bias [36].

To address the challenge of uncertainty in estimating the ATT with continuous propensity scores, several methodologies have been proposed in the literature. The most commonly used techniques include Nearest Neighbor Matching (NNM), Radius Matching (RM), Kernel Matching (KM), and Stratification Matching (SM). SM divides the range of the propensity score into intervals, ensuring that treatment and control units within each interval have similar propensity scores. However, SM may discard observations from blocks with no matching control units, whereas NNM ensures that each treated unit finds a match by comparing it to the nearest untreated unit in terms of propensity score. According to Becker and Ichino [37], NNM compares each treated subject to the untreated subject with the closest propensity score, providing robust estimations of the treatment effect.

## 3. Results

As demonstrated in Table 3, the farmers with animals infected with FMD and those with non-infected animals had distinct mean scores for various features. For instance, the formal education level of farmers with animals infected with FMD is much higher than that of farmers with non-infected animals. Additionally, those affected with FMD are more likely to have a smaller family size with subsidiary involvement from women. Considering other farm characteristics, farmers with animals afflicted with FMD have an IDR 570,642.230 higher household income and a TLU of cattle higher than 0.19 than farmers with non-infected animals. With communal cattle rearing, there were more cases of FMD than individual livestock sheds. Table 3 provides further details about the participants in this study. For example, the median age of the respondents was 54.10. The average formal education level was 8.53 years, exhibiting that the majority of farmers in Indonesia have not completed nine years of elementary education. Some responders have never been to a formal educational institution. The average farm household comprised 3.75 members, 1.27 TLU of cattle, 1 to 2 heads, and 513 m^2^ of land tenure. Most respondents are farmers with 20 years of experience in cattle fattening practices. It has been nearly two generations since the last incidence of FMD in Indonesia.

This study was conducted one month after the peak number of FMD cases (July 2022); therefore, most farmers have good knowledge, attitudes, and practices about FMD (Table 4). Animal healthcare workers have implemented disease management programs through disease socialization, vaccination, and cattle movement control. However, over half of all responders seem to have inadequate knowledge of FMD (Table 5). Most farmers are still not advised by officers. In contrast, most farmers have a positive attitude and value-based practices toward the FMD eradication program. Theoretically, this is because farmers have witnessed and suffered the losses caused by FMD. The losses incurred by FMD represent a significant portion of the rising costs associated with the prevention and control of FMD-infected cattle. According to Table 5, the average treatment cost per animal is IDR 316,548, while the average prevention cost is IDR 41,162. Prior to matching, the average prevention cost was IDR 41,778, while the treatment cost per animal remained unchanged. Furthermore, if the livestock cannot be rescued, the loss might escalate.

Table 6 presents the estimation of propensity scores using the Logit model (Equation (1)) to predict the likelihood of FMD infection. The resulting propensity score distribution for both infected and non-infected groups shows a common support region between 0.03079 and 0.64891, with an average score of 0.209 (Std. Dev = 0.136). Observations with propensity scores outside this common support range were excluded from the analysis. The results indicate that certain characteristics of smallholder farmers, such as inadequate land for cattle raising, are closely associated with the prevalence of FMD infection. Specifically, farmers who use cattle sheds with limited space are more vulnerable to FMD exposure, as virus-carrying agents can more easily spread in confined environments [44].

Most smallholder agricultural laborers are family based, typically consisting of a husband, wife, and their children. In this setup, the husband primarily works on the farm, while the wife occasionally assists with livestock care. As a result, farmers who involve women in the livestock-raising process tend to have a lower risk of FMD infection. Additionally, income from livestock sales is closely linked to the number of cattle a farmer owns. However, farmers with larger herds are more vulnerable to FMD, as increased movement of people in and out of the pen, often related to the sale or purchase of livestock, heightens the risk of disease transmission. A similar risk is observed in farmer cooperatives with communal pens. Due to the lack of control over animal movement, these pens are more susceptible to FMD. If one animal contracts the disease, it can quickly spread to the others. This suggests that specific characteristics of farmers, such as herd size and involvement in communal pens, may increase the susceptibility of their animals to FMD infection. Therefore, farmers at higher risk should be prioritized in FMD control programs.

We conducted several analyses to assess the presence of common support in both groups, ensuring the compatibility of the balancing test with the Logit model and verifying the reliability of the common support [26]. The results of the balance assessment using the NNM technique, presented in Table 7, indicate no statistically significant differences in the mean values of the independent variables between the two groups. This suggests that characteristics such as age, education, household size, women’s involvement in decision-making, land size, income, cattle ownership, farmer group, farming system type, and farming experience are balanced between farmers with FMD-infected animals and farmers with non-infected animals (Table 7). After confirming the equal distribution of the uncontrolled and control groups based on these variables (additional information related to residual balances, S1, Figure S1; S2, Figure S2; S3, Table S1), we proceeded to assess the ATT using the PSM method (Table 8). This allowed us to estimate the social and economic impacts of FMD outbreaks, including changes in livestock management practices and additional costs. The impact was measured using several alternative estimators, including SM, NNM, RM, and KM. A *t*-test was used as a control to illustrate the effects of FMD outbreaks before and after matching.

This study’s findings indicate that FMD outbreaks can alter farmers’ social behaviors, including knowledge, attitudes, and practice, and lead to extra expenses (Table 8). Farmers exposed to FMD compared with those without FMD exposure possess notable social and economic value differences. Farmers whose livestock are impacted by FMD are typically rated 1.4 to 1.9 points higher than those whose animals are unaffected. In addition, the costs borne by farmers affected by FMD exceed IDR 258,000 and IDR 270,000. This indicates that FMD has a social and economic impact on smallholder farmers. Additionally, a summary of the economic impact of FMD in selected countries is presented in Table 9.

## 4. Discussion

### 4.1. Livestock Management Practices in Response to FMD Prevalence

In general, farmers have limited knowledge of FMD (Table 5) in the studied area. As there had been no outbreaks of FMD for the past 36 years, farmers lack knowledge about the disease. This is further confirmed by the average livestock-raising experience of farmers, which is approximately 20.26 years (Table 4). Hence, farmer households lack experience in managing FMD outbreaks. This study was conducted during the FMD outbreak when the government incessantly conducted disease prevention socialization [52]. Therefore, some farmers may possess knowledge related to FMD outbreaks. Nevertheless, most farmer households are unaware of the risk of FMD transmission through the movement of farmers, animal products, and livestock equipment contaminated with disease outbreaks (Table 4).

Attitude is a readiness to respond positively or negatively to an object. The attitudes of farmer households are a response to the prevention, handling, and control of animal disease outbreaks [53]. Farmer households in this study demonstrated a positive attitude to FMD outbreak responses (Table 5). This attitude represents practicing proper biosecurity, getting vaccinated against diseases, seeking treatment, and consuming animal carcasses that have been afflicted by FMD outbreaks (Table 4). Practice is the implementation of the prevention, handling, and control of animal disease outbreaks. These practices include handling sick animals, products from infected animals, and carcasses of infected animals [54]. In this study, the farmers’ household practices were good overall. It can control FMD outbreaks and restrict widespread disease occurrence. Prevention practices are illustrated by many farmers participating in the government’s vaccination program and limiting access in and out of farms (Table 4). It is correlated to the farmers in Cambodia who will modify their disease management strategies by engaging in vaccination programs [55].

The average treatment costs per animal in the infected group were IDR 316,548, with the most considerable cost of treatment during infected livestock with an average of IDR 180,547. The cost of the treatment is not much different from farmers in Uganda, with a cost of USD 19 or around IDR 293,588 [50]. In contrast, the average treatment costs per animal in the non-infected group were IDR 41,162, with the most significant number for doctor’s examinations with an average of IDR 24,922.28.

### 4.2. Farmer Characteristics as Determinants of FMD Prevalence

The estimation results (Table 5) indicate that the socioeconomic characteristics of farmers are strongly related to the risk of infection from strategic infectious animal diseases. In particular, land size affects the livestock infection rate. This finding also confirms that small land size causes livestock population density, increasing the risk factor of livestock being infected with the disease. The land size also impacts the proximity of farmers’ cages to other breeders, which means that transmission can more easily occur [56]. The close proximity of farmers’ cages is due to farmers being members of livestock groups. As a result of high farm density and reliance on community grazing, smallholder systems are troublesome due to their multiple inter-farm connections.

Moreover, farmers may have fewer clear incentives to combat the disease. Vaccinating smallholdings has logistical challenges in achieving high coverage, particularly in countries with less proficient veterinary services that lack the capacity to enforce standards and provide compensation. Consequently, there is a requirement for FMD control in smallholder systems [19]. If achieving universal control is not feasible, farmers desiring to manage FMD should be supported through the provision of high-quality vaccinations (in case doubts arise about their effectiveness, vaccine acceptance may be limited) and by mitigating the harm caused by individuals not participating in FMD management. Governments in certain nations subsidize vaccinations for small farms. Although not yet a possibility, commodity-based commerce would give businesses access to affluent markets without the massive obstacle of establishing national or zonal independence [55].

The findings confirm women’s role in strategic infectious animal control in livestock businesses. The role of women contributes to the decision-making process related to livestock rearing and the costs of running a livestock business [57]. The role of women in the livestock industry is access to, control over, and advantages over available resources. In accessing information sources, they have less access than men. This is because women are restricted in mobility by their partners [58]. In the control aspect, the role of men dominates in the livestock business, but in its implementation, it still involves women as laborers and influences the livestock business that is practiced. Regarding benefits, the livestock business activities carried out benefit all family members [59]. The majority of smallholder farmers use their livestock as a source of income and a depository for their assets, which are frequently managed in conjunction with other means of subsistence [60]. Increased livestock ownership increases the likelihood of cattle infection, particularly infectious illnesses such as FMD. If cattle become contaminated with FMDV, non-susceptible animals pose a danger of FMD transmission. The risk of contamination is greater close to infected buildings. The presence of FMD on a property may be identified (“infected premises”) or yet, undetected [44].

### 4.3. Impact of FMD on Farmers’ Socioeconomic Conditions

The results (Table 8) demonstrate that the average impact of FMD on socioeconomics is almost identical and significant at one percent with the SM, NNM, RM, and KM methods. The outbreak of FMD can have social consequences that are evident at the individual, household, and community levels. The social impacts on farmer households have been demonstrated through the KAP approach (Table 3). FMD can significantly impact the social knowledge, attitudes, and practices of breeders. FMD is an extremely infectious viral illness that impacts animals with divided hooves, like cattle, resulting in substantial losses in livestock productivity and trade. As a result, breeders may experience economic losses, leading to changes in their attitudes toward breeding and animal husbandry practices. They may also be forced to adopt new measures to prevent the spread of FMD, such as increased biosecurity measures, leading to changes in their knowledge and practices. Moreover, FMD outbreaks may result in changes in public attitudes toward livestock farming and consumption, potentially affecting the breeders’ reputation and ability to sell their products [55].

The impact of FMD on the per capita expenditure of farmers for treatment costs ranges from IDR 258,000 to IDR 270,000 more than farmers with non-infected animals. In absolute terms, the SM method shows the most significant impact (IDR 270,000), and the RM method shows the most negligible impact (IDR 258,000) per capita expenditure. FMD can have a considerable impact on medical expenses. It can include medical expenses and biosecurity improvement costs. The economic losses caused by FMD outbreaks can also affect farmers’ ability to pay for medical expenses. Therefore, the control and prevention of FMD are essential to reduce medical expenses and maintain the health and productivity of farm animals. Since euthanasia of FMD-positive animals is not a mandatory policy, animals that survive FMD may still be sold, contingent upon a thorough veterinary assessment prior to slaughter. Therefore, the costs incurred by farmers for depopulation require government attention and support. The government, through its extension officers, can support farmers by providing education on disease management, assisting with the isolation of infected livestock, and offering compensation for animals that succumb to the disease [43,61,62,63]. The Government of Indonesia regulates this in ministerial decree no. 518/KPTS/PK.300/M/7/2022, which consists of instructions for the depopulation of healthy animals, sick animals, suspected illnesses, or animals carrying FMD [64]. These measures include regular veterinary inspections, vaccination campaigns, and quarantine protocols to prevent the spread of diseases. The government has stipulated the amount of assistance in an oral and nail disease emergency in decree no. 08048/KPTS/PK.300/F/07/2022, which states that farmers with cattle affected by FMD that die or are euthanized will be given cash of IDR 10,000,000. This assistance is limited to five animals per ownership [65].

Table 9 outlines the economic impact of Foot and Mouth Disease (FMD) across various countries, emphasizing the broader implications of the disease. In regions with endemic FMD, the disease significantly disrupts production, trade, and control measures, with the poorest populations suffering the most due to limited market access. In countries like Ethiopia, India, and Uganda, smallholder farmers experience heightened vulnerability, as the disease impacts milk production, livestock health, and market value, often leading to financial distress. The social and psychological effects are particularly severe in countries with intensive farming systems, such as India. While some nations, like Nigeria, benefit from effective control measures that provide a high return on investment, the overall economic burden remains substantial. In developed countries like Australia, the impact of FMD is largely due to trade disruptions and production losses, which can be exacerbated by trade sanctions. These findings underscore the need for targeted FMD control strategies, particularly for smallholders in developing regions, to mitigate economic losses and enhance food security.

The increasing production costs, particularly the treatment costs associated with FMD outbreaks, can have significant broader implications for cattle farmers’ household income and food security. FMD treatment typically involves direct costs for veterinary services, medications, and potentially the culling of infected animals, all of which place a financial strain on farmers, especially those with limited resources [66,67]. For smallholder cattle farmers, who often rely on livestock as their primary source of income, the economic impact of FMD can be severe. The direct financial burden of treatment can deplete savings or reduce farmers’ ability to invest in other necessary areas, such as improving farm infrastructure or purchasing additional livestock. Moreover, the loss of cattle, either due to disease or culling, directly impacts household income, reducing the farmer’s capacity to meet daily living expenses or reinvest in their farming operations [68].

In terms of food security, FMD outbreaks can disrupt the supply of meat, which is a crucial source of nutrition in rural areas. With the loss of livestock and the high costs associated with treating infected animals, farmers may be forced to sell remaining cattle at lower prices, further compromising their financial stability [69]. This can lead to a reduced ability to provide for the household’s nutritional needs, exacerbating food insecurity [70]. Thus, the increased treatment costs associated with FMD outbreaks not only threaten the financial stability of cattle farmers but also have long-term consequences for household income and food security, making it critical for policymakers to address both the economic and social dimensions of FMD prevention management.

Our findings have significant policy implications for Indonesia and globally, given that animals, equipment, transportation, and human movement can transmit the FMD virus. Implementing effective FMD mitigation strategies through comprehensive risk management is crucial. This approach involves several key actions, such as controlling animals as a source of disease (reservoir), identifying high-risk areas for virus outbreaks, and addressing risky practices that facilitate virus transmission [71,72,73]. Specifically, targeted control measures could involve providing additional resources and training to high-risk farmers, particularly those with large herds or in communal farming environments. These farmers could benefit from focused educational campaigns on biosecurity practices, vaccination protocols, and disease surveillance to minimize the risk of infection.

Further, the establishment of a robust monitoring system to track livestock movements and animal health status would help identify areas most vulnerable to outbreaks [71,73]. Closing affected areas to the movement of animals, products, and equipment is another practical measure to prevent the spread of the virus. In this context, local authorities could implement checkpoints for livestock transportation, ensuring that only disease-free animals are allowed to enter or leave designated outbreak zones. Moreover, previous studies have highlighted that cattle farmers in Yogyakarta are particularly susceptible to FMD risks [74]. To mitigate these risks, targeted support, such as financial assistance for subsidies for vaccines or veterinary services, could help high-risk farmers better manage the costs associated with FMD prevention.

Overall, our results suggest that FMD outbreaks have impacted the socioeconomic conditions of smallholder cattle farmers in Yogyakarta. After nearly four decades, the re-emergence of FMD cases in Indonesia has impacted the social and economic aspects of livestock farming [75,76,77,78]. The impact of FMD disease extends beyond the scope of animal health to include socioeconomic factors. Farmers impacted by FMD incur significant losses, including increased costs for livestock treatment, decreased livestock production, declining livestock prices, and high livestock mortality rates [78].

## 5. Conclusions

The findings of this study are highly relevant not only for Indonesia but also in a broader international context—particularly considering that Foot and Mouth Disease (FMD) will reappear in Europe in 2025. This unexpected resurgence in a region previously considered free from FMD highlights how transboundary animal diseases remain a global threat. It also emphasizes the importance of understanding how farmers in different parts of the world experience and respond to such outbreaks, especially those operating in vulnerable, small-scale farming systems.

In our work, we explored the key characteristics that influence a farmer’s likelihood of experiencing an FMD outbreak and assessed how the disease affects the social and economic realities of smallholder cattle farmers. We found that several farmer-specific factors, such as household income, participation in farming groups, the number of cattle owned, and particularly the involvement of women in decision-making are closely linked to the risk of FMD infection. This suggests that some farmers, due to their socioeconomic conditions, face a higher vulnerability to the disease. Beyond identifying these risk factors, our results show that FMD significantly changes farmers’ behaviors and increases their financial burden. Social behaviors, specifically knowledge, attitudes, and practices related to animal health, improved significantly among farmers with FMD-infected animals, likely as a reaction to the crisis. Economically, those affected by FMD had to spend between IDR 258,000 and IDR 270,000 more per animal on treatment compared to their non-affected peers. Based on these insights, we recommend several targeted policy measures: (1) FMD control and prevention strategies should be tailored for high-risk farmers, especially those with limited land, greater herd sizes, higher incomes, or those who practice communal cattle rearing. These strategies should include improved training, tailored vaccination plans, and access to subsidized disinfection resources. (2) Treatment-related financial pressures could be eased through compensation schemes that more accurately reflect real losses and through the creation of emergency funds or microinsurance products designed for smallholders. (3) More effective education programs are needed to address knowledge gaps. (4) We suggest that the positive role women play in reducing FMD risk should be encouraged and supported through gender-sensitive training, peer networks, and inclusive extension services. Together, these actions could not only strengthen Indonesia’s ability to manage FMD but also provide valuable guidance for other regions. Supporting the resilience of smallholder farmers is not just a local priority but a global necessity in the face of re-emerging animal health threats.

In addition, this study has several limitations that should be noted. The findings are based on a specific region, which may not fully represent other areas with different socioeconomic conditions. Additionally, this study focuses on short-term impacts, while the long-term effects on livelihoods and local economies remain underexplored. Future research should expand geographically, incorporate longitudinal data, and examine the broader socioeconomic consequences, including food security and community well-being. Moreover, while this study highlights the role of women in managing FMD risks, further research on gender-sensitive interventions and their effectiveness in diverse contexts is needed. This will contribute to a more comprehensive understanding of FMD’s long-term impacts and inform more effective policy and intervention strategies.

## Figures and Tables

**Table 1:** Distribution of the respondent sample.

Region	Frequency
Kulon Progo	176
Bantul	212
Gunungkidul	496
Sleman	108
Total	992

**Table 2:** Definition of variables.

Variable	Definition and Measurement	Category
Outcome variables		
Social	KAP	Score (continuous)
Economic	Treatment cost ^a^	IDR
Dependent variable		
Effect	Cattle infected with FMD	Dummy (1 = yes; 0 = otherwise)
Independent variables		
Age	Age of the farmers	Years (continuous)
Education	Formal education level	Years (continuous)
Household size	Size of household	Number of people (continuous)
Land size	The total area of land managed by the farmers	m^2^
Women’s involvement in decision-making	Women’s involvement in decision-making	Dummy (1 = yes; 0 = otherwise)
Income	The total family revenue for the months surveyed	Indonesian rupiah (IDR)
Farmer group	Member of a farmers’ organization	Dummy (1 = yes; 0 = otherwise)
Cattle ownership	The total number of cattle kept by the farmers	TLU ^b^ (continuous)
Farming system types	The type of beef cattle business ^c^	Categoric
Farming experience	Cattle farmers’ experience	Years (continuous)

^(a)^ ATC $=1/n\sum_{1}^{N}\left(F+M+I\right)$, where ATC is the average treatment costs per animal (IDR); F is fees for veterinarians/farmers (IDR); M is the cost of medicines (vaccines)/farmer (IDR); I is the cost of indigenous treatment during the infected period (IDR); N is the number of farmers; and n is the total number of animals infected. ^(b)^ TLU stands for Tropical Livestock Unit, representing a standardized measure of 250 kg of live animal weight [34]. ^(c)^ 1 = fattening; 2 = rearing; and 3 = breeding.

**Table 3:** Descriptive statistics of farmer characteristics.

Variable	Total Mean (S.E.)	Min	Max	Group’s Mean		
				Infected (*n* = 202)	Non-Infected (*n* = 790)	Differences (S.E.)
Age	54.10 (0.411)	18	85	53.71 (0.862)	54.19 (0.467)	0.481 (1.022)
Education	8.53 (0.117)	0	19	9.27 (0.275)	8.34 (0.128)	−0.929 ** (0.289)
Household size	3.75 (0.047)	1	9	3.56 (0.094)	3.8 (0.054)	0.234 * (0.117)
Land size	513.11 (33.739)	6	9024	391.81 (47.496)	544.12 (40.526)	152.316 (83.684)
Women’s involvement in decision-making	0.76 (0.014)	0	1	0.62 (0.034)	0.8 (0.014)	0.172 ** (0.033)
Income	1,681,540.59 (46,077.21)	250,000	9,800,000	2,135,983.50 (119,115.41)	1,565,341.26 (48,376.19)	−570,642.230 ** (113,033.81)
Farmer group	0.59 (0.016)	0	1	0.87 (0.024)	0.51 (0.018)	−0.352 ** (0.037)
Cattle ownership	1.27 (0.028)	0	9	1.42 (0.069)	1.23 (0.030)	−0.192 * (0.069)
Farming system types	2.55 (0.024)	1	3	2.49 (0.054)	2.56 (0.026)	0.076 (0.059)
Farming experience	20.26 (0.488)	1	75	20.51 (1.028)	20.19 (0.554)	−0.319 (1.213)

* *p* < 0.05. ** *p* < 0.001.

**Table 4:** Frequency table for KAP score answers for smallholder farmers relating to FMD.

Elements		Response (%)	
		Yes	No
Knowledge			
1.	Do you know the FMD-affecting agent?	812 (81.85)	180 (18.15)
2.	The symptoms of FMD do not include salivary secretion, gum lesions, nail lesions, or udder lesions.	486 (48.99)	506 (51.01)
3.	The FMD cannot kill your livestock.	493 (49.70)	499 (50.30)
4.	Can FMD generate significant economic losses?	903 (91.03)	89 (8.97)
5.	Methods of prevention, such as vaccination, isolation, and biosecurity, cannot be employed to prevent FMD.	635 (64.01)	357 (35.99)
6.	Do you have any information on the transmission of FMD?	541 (54.54)	451 (45.46)
7.	FMD is transmissible through the death of diseased animals, secretions, spores, and humans.	518 (52.22)	474 (47.78)
8.	FMD can be transferred between animals and humans (zoonosis).	300 (30.24)	692 (69.76)
9.	Biosecurity is a precaution used to prevent the spread of FMD.	94 (9.48)	898 (90.52)
10.	Bacterial spores can survive for many years in the soil.	271 (27.32)	721 (72.68)
11.	Animal products such as meat, milk, and processed foods can potentially spread FMD.	501 (50.50)	491 (49.50)
Attitudes			
1.	The FMD epidemic is hazardous.	880 (88.71)	112 (11.29)
2.	Vaccinations cannot be used to boost the immune system.	436 (43.95)	556 (56.05)
3.	Biosecurity practices on farms, such as disinfectants, visitation control, and movement restrictions, can reduce FMD transmission.	883 (89.01)	109 (10.99)
4.	Treatment and isolation of infected animals help control FMD.	907 (91.43)	85 (8.57)
5.	The transmission of FMD can be controlled by burning deceased animals instead of slaughtering suspected livestock.	635 (64.01)	357 (35.99)
6.	Consuming meat, milk, and processed products from infected animals is potentially hazardous.	747 (75.30)	245 (24.70)
Practices			
1.	Are you concerned with the treatment and disease control program on your farm?	528 (53.23)	464 (46.77)
2.	Do you use disinfectants to sterilize the farmland’s surroundings?	615 (62.00)	377 (38.00)
3.	Do you cremate or bury FMD-infected animals?	626 (63.10)	366 (36.90)
4.	Do you treat sick animals using indigenous treatments such as herbal medicine?	441 (44.46)	551 (55.54)
5.	Do you seek treatment at a veterinary facility if FMD is suspected?	852 (85.89)	140 (14.11)
6.	Do you vaccinate livestock to prevent disease?	754 (76.01)	238 (23.99)
7.	Do you wash your hands and boots after handling an animal?	934 (94.15)	58 (5.85)
8.	Has a drainage system been installed on your farm?	707 (71.27)	285 (28.73)
9.	Do you control employee or visitor access to your farm?	805 (81.15)	187 (18.85)

**Table 5:** Cross-tabulation of farmers’ socioeconomic conditions by FMD prevalence.

Variables			Infected	Non-Infected	Total
Social					
Knowledge	Less	Freq.	69	508	577
		%	34.20	64.30	58.17
	Good	Freq.	133	282	415
		%	65.80	45.70	41.83
Attitude	Negative	Freq.	39	135	174
		%	19.30	17.10	17.54
	Positive	Freq.	163	655	818
		%	80.70	82.90	82.46
Practice	Less	Freq.	11	280	291
		%	5.40	35.40	29.33
	Good	Freq.	191	510	701
		%	94.60	64.60	70.67
Economic					
Treatment costs (IDR)			316,548	41,162	

**Table 6:** Logit model results of sociodemographic determinants influencing the probability of FMD outbreaks.

Variable	Coefficient	S.E.	Z-Value	*p* > |z|
Age	−0.0019444	0.004	−0.39	0.696
Education	0.0204105	0.015	1.36	0.175
Household size	−0.0491321	0.036	−1.34	0.179
Land size	−0.000165	0.000	−2.66	0.008 **
Women’s involvement in decision-making	−0.3105893	0.107	−2.89	0.004 **
Income	0.000101 × 10^−3^	0.000	3.02	0.003 **
Farmer group	0.8431656	0.116	7.24	0.000 **
Cattle ownership	0.0842355	0.052	1.61	0.09 *
Farming system types	0.0048139	0.063	0.08	0.939
Farming experience	0.0038256	0.003	1.03	0.304
Constant	−1.392829	0.428	−3.25	0.001 **
Pseudo R^2^	0.1251			
LR chi^2^ (10)	125.45			
Prob > chi^2^	0.0000			
Observations	958			

* *p* < 0.1. ** *p* < 0.01.

**Table 7:** Conformity evaluation of the common supports after matching.

Variable	Mean		*t*-Test ^a^		Bias (%)
	Infected (*n* = 202)	Non-Infected (*n* = 756)	T	*p* > |t|	
Age	53.71	54.16	−0.36	0.72	−3.5
Education	9.27	9.09	0.49	0.63	4.9
Household size	3.56	3.54	0.14	0.89	1.4
Land size	391.81	369.59	0.31	0.76	2.4
Income	2,100,000	2,000,000	0.93	0.35	9.8
Cattle ownership	1.42	1.36	0.66	0.51	7.2
Farming experience	20.51	20.86	−0.24	0.81	−2.3

^a^ The non-significant results confirm that the means of the infected and non-infected groups are equal.

**Table 8:** The socioeconomic impact of FMD on smallholder farmers.

Outcome	Before Matching ^a^	Average Treatment of the Treated (ATT) ^b^			
		SM	NNM	RM	KM
Social	1.43034548 ** (0.63)	1.431 ** (0.60)	1.673 ** (0.85)	1.977 ** (0.70)	1.583 ** (0.60)
Economic	265,349.505 ** (26,951.96)	270,000 ** (25,154.95)	265,000 ** (26,951.96)	258,000 ** (27,910.59)	269,000 ** (24,663.81)

^a^ Mean difference between infected and non-infected groups. ^b^ “psmatch2” in the STATA 14 command was employed to execute the ATT estimates [45]. Standard errors are represented by the numbers in parentheses. ** *p* < 0.01.

**Table 9:** The economic impact of FMD in selected countries.

Country	Estimated Losses	Main Losses	Notable Impacts
Global	USD 6.5–21 B (endemic); >USD 1.5 B (free)	Production, control, trade	The poorest are the most affected; market access [19]
Ethiopia	USD 0.9 M/year; USD 11/case	Milk, draft power, fertility	Losses are higher in intensive systems [46]
India	USD 208–1008/animal	Distress sale, mortality, milk	High social/psychological impact [47]
Nigeria	USD 16 M total	Production, treatment	High benefit–cost ratio for control [48]
Australia	AUD 10–85 B (scenario-based)	Trade, production	Losses depend on trade sanctions [49]
Uganda	USD 196–1552/herd (salvage sale)	Milk, beef, market value	Smallholders most affected [50]
Malaysia	MYR 390.24/herd	Mortality, weight loss	Breed-specific impacts [51]

## Data Availability

The data are contained within this article and Supplementary Materials.

## Supplementary Materials

The following supporting information can be downloaded at https://www.mdpi.com/article/10.3390/vetsci12060542/s1, S1. Figure S1: Covariate distributions for infected and non-infected groups before and after matching; S2. Figure S2: Propensity score distribution overlap for treated and control groups; S3, Table S1: Balance statistics for covariates before and after matching.
