# Supplementary material for: Socioeconomic Impact of Foot and Mouth Disease Outbreaks on Smallholder Cattle Farmers in Yogyakarta, Indonesia
Source: Vet Sci. 2025 Jun 3;12(6):542. doi: 10.3390/vetsci12060542 (PMC12197744; doi:10.3390/vetsci12060542)
Supplement: Supplementary file 1 [file vetsci-12-00542-s001.zip › Supplementary 1 (Figure S1).pdf]

Figure S1. Covariate Distributions for Infected and Uninfected Groups Before and After Matching

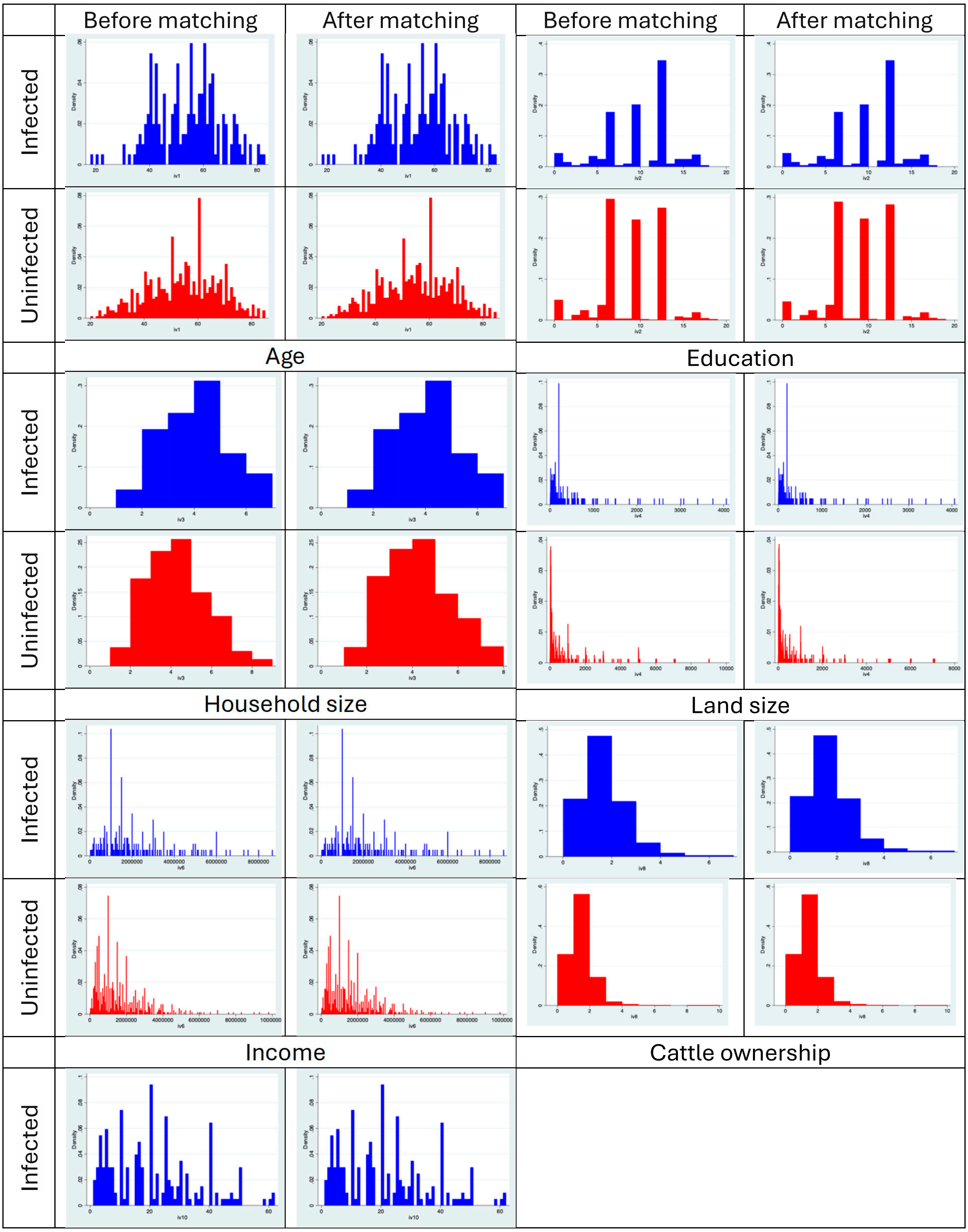

Uninfected	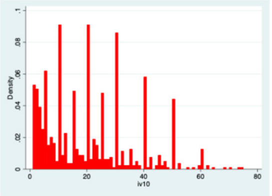	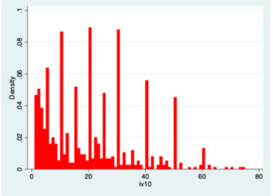	
	Farming experience		
