# Supplementary material for: Socioeconomic Impact of Foot and Mouth Disease Outbreaks on Smallholder Cattle Farmers in Yogyakarta, Indonesia
Source: Vet Sci. 2025 Jun 3;12(6):542. doi: 10.3390/vetsci12060542 (PMC12197744; doi:10.3390/vetsci12060542)
Supplement: Supplementary file 1 [file vetsci-12-00542-s001.zip › Supplementary 3 (Table S1).pdf]

Table S1. Balance Statistics for Covariates Before and After Matching

Variables	Standardized differences		Variance ratio	
	**Raw**	**Matched**	**Raw**	**Matched**
Age	-0.037	-0.023	0.870	0.898
Education	0.247	0.113	1.174	1.085
Household size	-0.164	0.032	0.766	0.845
Land size	-0.162	0.027	0.351	0.797
Women decision	-0.386	0.030	1.451	0.985
Income	0.371	-0.011	1.550	1.005
Farmer group	0.823	-0.029	0.465	1.067
Cattle ownership	0.209	0.118	1.330	0.771
Farming system types	-0.100	0	1.084	0.967
Farming experience	0.021	0.014	0.879	0.901
