# Supplementary figures and images for: Socioeconomic Impact of Foot and Mouth Disease Outbreaks on Smallholder Cattle Farmers in Yogyakarta, Indonesia
Source: Vet Sci. 2025 Jun 3;12(6):542. doi: 10.3390/vetsci12060542 (PMC12197744; doi:10.3390/vetsci12060542)

Figure S2. Propensity Score Distribution Overlap for Treated and Control Groups

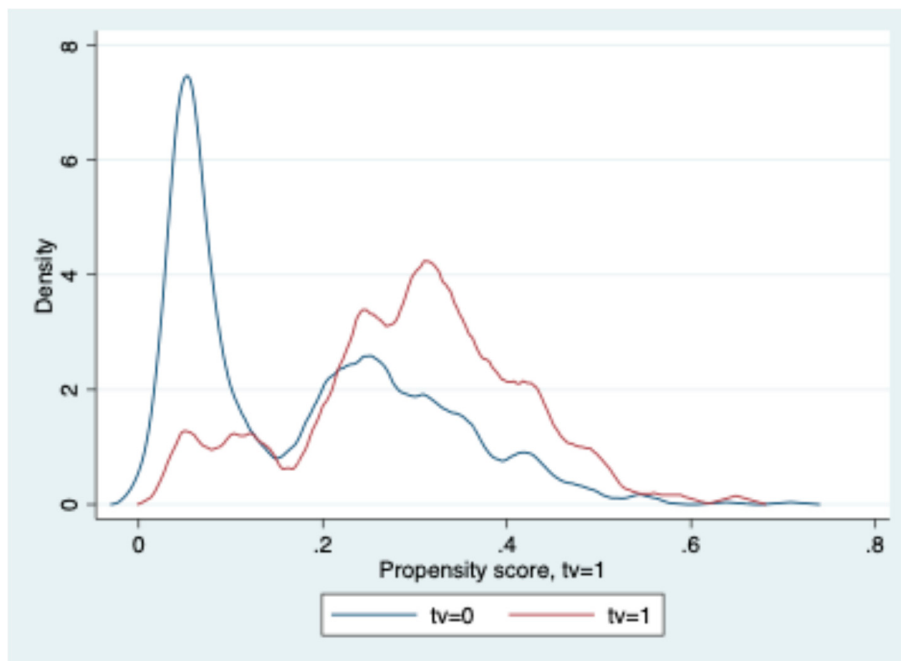

Supplement: Supplementary file 1 [file vetsci-12-00542-s001.zip › Supplementary 2 (Figure S2).pdf]
